# *Pueraria montana* Population Structure and Genetic Diversity Based on Chloroplast Genome Data

**DOI:** 10.3390/plants12122231

**Published:** 2023-06-06

**Authors:** Jiahui Sun, Yiheng Wang, Ping Qiao, Lei Zhang, Enze Li, Wenpan Dong, Yuping Zhao, Luqi Huang

**Affiliations:** 1 State Key Laboratory Breeding Base of Dao-di Herbs, National Resource Center for Chinese Materia Medica, China Academy of Chinese Medical Sciences, Beijing 100700, China; sunjh_2010@sina.com (J.S.); wangyiheng1991@foxmail.com (Y.W.);; 2 Key Laboratory of Biology and Cultivation of Herb Medicine, Ministry of Agriculture and Rural Affairs, Beijing 100700, China; 3 China Academy of Chinese Medical Sciences, Beijing 100700, China; 13512005456@126.com; 4 Laboratory of Systematic Evolution and Biogeography of Woody Plants, School of Ecology and Nature Conservation, Beijing Forestry University, Beijing 100083, China; lienze@bjfu.edu.cn

**Keywords:** *Pueraria montana*, population structure, genetic diversity, chloroplast genome, divergence time estimation

## Abstract

Despite having a generally conserved structure, chloroplast genome data have been helpful for plant population genetics and evolution research. To mine *Pueraria montana* chloroplast genome variation architecture and phylogeny, we investigated the chloroplast variation architecture of 104 *P. montana* accessions from across China. *P. montana*’s chloroplast genome showed high diversity levels, with 1674 variations, including 1118 single nucleotide polymorphisms and 556 indels. The intergenic spacers, psbZ-trnS and ccsA-ndhD, are the two mutation hotspot regions in the *P. montana* chloroplast genome. Phylogenetic analysis based on the chloroplast genome dataset supported four *P. montana* clades. *P. montana* variations were conserved among and within clades, which showed high gene flow levels. Most *P. montana* clades were estimated to have diverged at 3.82–5.17 million years ago. Moreover, the East Asian summer monsoon and South Asian summer monsoon may have accelerated population divergence. Our results show that chloroplast genome sequences were highly variable and can be used as molecular markers to assess genetic variation and relationships in *P. montana*.

## 1. Introduction

Chloroplasts are maternally inherited in most angiosperms and have their own genome. The chloroplast genome is circular, ranging from 107 to 218 kb among the photosynthetic plant species [1]. Comparative genomic architecture studies have shown that chloroplast genome structures, gene numbers, and gene content organization are highly conserved among most land plants. The typical chloroplast genome contains two inverted repeats (IR) regions that separate large (LSC) and small single-copy (SSC) regions. The chloroplast genome encodes 120–130 genes that primarily participate in photosynthesis, transcription, and translation [2].

The chloroplast genome’s evolution rate is moderate compared with nuclear or mitochondrial genomes, and the ratio of mutation rates in the mitochondrial, chloroplast, and nuclear genes is about 1:3:10 in seed plants [3]. The chloroplast genome’s protein-coding gene sequences are widely used for phylogenetic analysis at high taxonomic levels, such as family or order levels [4,5,6,7,8]. Moreover, whole chloroplast genome sequences are popular molecular markers for reconstructing relationships and species identification due to their variability among interspecies levels [9,10,11]. For example, the coding regions of maturase K (matK), ribulose bisphosphate carboxylase large subunit (rbcL), and ycf1 are used as DNA barcodes for plants [12,13]. Similarly, spacer regions trnH-psbA, rpl32-trnL, rps16-trnQ, and rbcL-accD have a long history in plants evolution analysis [14,15].

Chloroplast genome sequences have been widely used as the DNA marker for genetic diversity research with the development of next-generation sequencing (NGS) methods. Previous studies have provided many genetic resources for single sequence repeats (SSRs), single nucleotide polymorphisms (SNPs), and insertion/deletion polymorphisms (indels) in chloroplast genomes at the intraspecies level. Chloroplast genetic resources have been used to examine genetic divergence within endangered or medicinal species [16,17,18], biogeographical structure [19,20,21,22], gene flow among subpopulations [23,24], and cultivar origins and domestication [25,26,27]. Maternally inherited chloroplast-genome-based evolutionary studies must sometimes be complemented by nuclear genomic information.

*Pueraria* Candolle includes ~20 species distributed in Southeast Asia [28]. Lee and Hymowitz (2001) were first to use molecular methods to confirm polyphyly within Pueraria, although they concluded that a “more rigorous molecular investigation” was required to definitively comprehend its evolutionary history. Egan et al. (2016) further confirmed polyphyly within Pueraria s. l., and all analyses reveal five distinct clades defined as five genera throughout the Phaseoleae tribe—*P. wallichii*, *P. stricta*, *P. phaseoloides*, *P. peduncularis* + *P. yunnanensis*, and the remaining species grouped into the main clade of *Pueraria*, called *Pueraria sensu stricto* (*Pueraria s. str.*) [29]. And *Pueraria* s. s. is sister to Psoraleeae. Some *Pueraria* species located in *Pueraria s. str.*, especially for Pueraria montana (Lour.) Merr. (kudzu), have been used to treat medical ailments and as textile, food, and paper sources since 500 BC in China. The *P. montana* was estimated to have separated from its closely related species at around 4.63 million years ago (mya). It has been divided into three varieties: var. *lobata* (Willd.) Maesen & S.M.Almeida ex Sanjappa & Predeep, var. *montana*, and var. *thomsonii* (Benth.) M.R.Almeida. However, these three varieties are notoriously difficult to identify morphologically due to overlapping taxonomic characteristics, such as the flower size and terminal leaflet shape, which are the predominant characteristics for distinguishing them [28,30]. Recent molecular data supported the three varieties forming a monophyletic group [29]; however, the three varieties did not form as separate clades [31]. *P. montana* var. *lobata* is a noxious invasive species in North America, first introduced at the 1876 Centennial Exposition in Philadelphia [32].

Previous studies have explored *P. montana*’s genetic diversity using various markers, including allozymes [32,33], random amplified polymorphic DNAs (RAPDs) [34], sequence-related amplified polymorphisms [35], inter-simple sequence repeats (ISSRs) [36,37,38], and microsatellites [39,40,41]. However, few studies have examined its population structure and genetic diversity based on DNA sequence markers. With their high reproducibility, DNA sequence markers increase our likelihood and ability to detect genetic variation [42]. *P. montana*’s chloroplast and nuclear genomes have been sequenced [43,44,45], enabling their use for genetic and evolutionary studies.

This study sequenced the chloroplast genomes of 70 *P. montana* accessions from mostly wild distributions in China. The complete chloroplast genome was assembled to identify sequence variations and diversity among accessions. Phylogenetic, population structure, and principal component analyses were used to investigate genetic divergence among *P. montana* accessions and identify their subclades. Furthermore, we assessed genetic divergence and distance among subclades. These results provide a further case study for discovering chloroplast genome mutation rates at the intraspecies level and a genetic variation resource for breeding this ecologically and economically important species, and management of invasive species.

## 2. Results

### 2.1. Pueraria Montana Plastid Genome Assembly

This study used genome skimming methods to assemble complete chloroplast genomes for 70 *P. montana* accessions with high mean coverage (120×). The *P. montana* chloroplast genomes were circular molecules with a typical quadripartite structure and sizes of 149,452–154,595 bp, including two IRs of 23,678–25,655 bp, separated by an LSC of 84,055–85,316 bp and SSC of 17,957–18,017 bp. Their overall GC content was 35.3%–35.4%. *P. montana*’s chloroplast genome encodes 124 genes, including 87 protein-coding genes, 29 transfer RNA (tRNA) genes, and 8 ribosomal RNA (rRNA) genes. The 87 protein-coding genes are divided into self-replicated, photosynthesis-related, and other types. All annotated chloroplast genomes were deposited in GenBank (Table S1).

### 2.2. Chloroplast Genome Sequence Variation in Pueraria Montana

**Figure 1:**
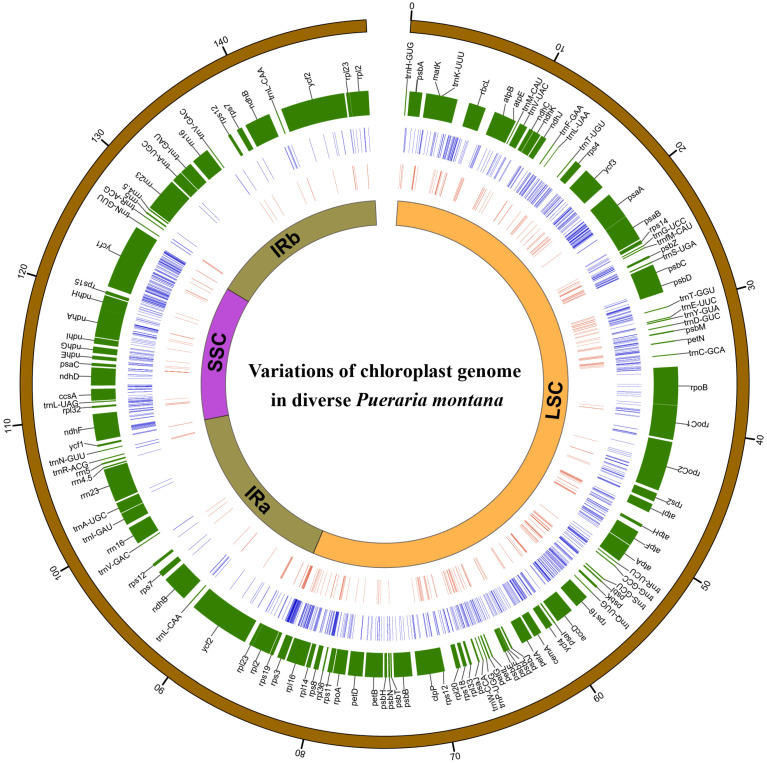
The overall distribution of SNPs and indels across the *P. montana* chloroplast genome. A circular map showing the chloroplast genome structure with the outer distance shown in kb. All genes (including protein coding, tRNAs, and rRNAs) and their locations are shown in the second circle. The third and fourth circles show SNPs and indels identified across all 104 accessions, respectively.

Combining these *P. montana* chloroplast genomes with those from GenBank, there were 104 accessions in total (Table S1). The alignment length was 158,274 bp, and 1674 variations, including 1118 SNPs and 556 indels, were identified in the *P. montana* chloroplast genomes (Figure 1). The 1118 SNPs included 615 singleton and 503 parsimony-informative sites. The average SNP density was 10.46/kb across the entire chloroplast genome, 9.26/kb in the LSC, 13.28/kb in the SSC, and 1.7/kb in the IR region. The overall π was 0.00055. The IR region had a lower SNP-based genetic diversity than the LSC and SSC regions. When π was averaged over 800 bp windows, two intergenic spacers (psbZ-trnS and ccsA-ndhD) had the highest sequence divergence (Figure S1). We identified 556 indels in the chloroplast genomes of the 104 *P. montana* accessions, most located in noncoding regions.

### 2.3. Pueraria Montana Phylogeny based on the Chloroplast Genome

**Figure 2:**
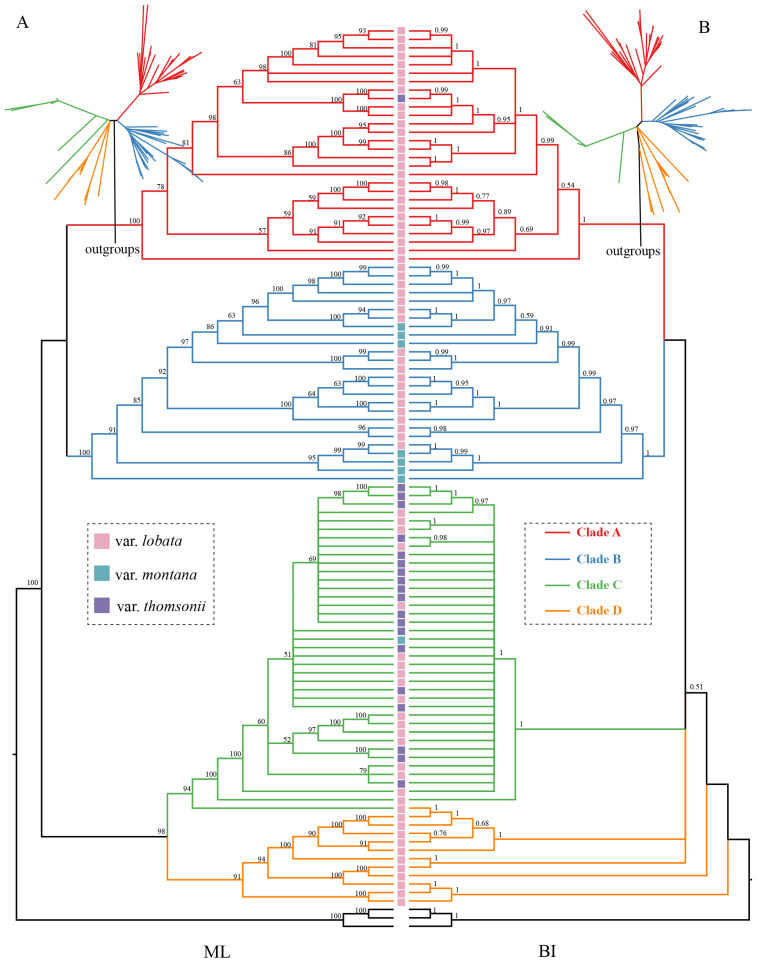
A tanglegram phylogenetic analysis using (**A**) ML and (**B**) BI trees to illustrate the relationships among *P. montana* accessions. Different colored squares represent different variants.

We used maximum likelihood (ML) and Bayesian inference (BI) methods to construct phylogenetic trees based on entire chloroplast genome sequences (Figure 2). A tanglegram was used to compare ML and BI trees, showing that the overall phylogenetic structure and clustering of the accessions in the two trees were nearly identical (the same accession in two trees can connect with itself at the same location in the clusters). Both results supported the separation of the 104 accessions into 4 clades (Figure 2). The resulting network with 4 subclades is shown in Figure S2.

Twenty-eight accessions from Anhui, Chongqing, Gansu, Guangdong, Henan, Hubei, Jiangsu, Jilin, Liaoning, Shaanxi, Shandong, Shanxi, and Yunnan formed Clade A, including 27 haplotypes. Clade B contained 26 samples, including 24 haplotypes. Clade A was sister to Clade B with high support values in both ML and BI trees. Clade C included the most samples with 39 accessions, containing 26 samples from GenBank, including 2 varieties of var. thomsonii and var. lobata. The ML tree supported Clade C being a sister to Clade D with 98% support. However, the BI tree did not support Clade D as a separate clade. Clade D included 11 samples from Gansu, Shandong, Shanxi, Sichuan, Jiangsu, Zhejiang, Shaanxi, Hubei, and Hebei. Clades C and D had 10 and 11 distinct haplotypes, respectively.

### 2.4. Population Structure and PCA of Pueraria Montana

**Figure 3:**
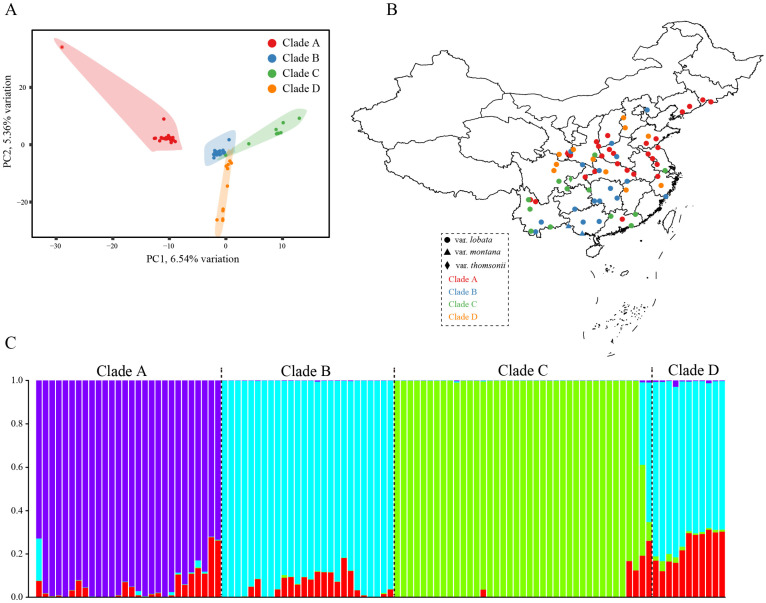
Population structure and PCA of all 104 accessions. (**A**) PCA of all accessions. (**B**) The position of 70 collected *P. montana* accessions (**C**) Population structure clustering with the optimal K (4).

A PCA based on the entire chloroplast genome sequences showed four significant principal components (PCs; Figure 3A). The first two PCs only explain >11.9 % of the total variance. The first PC significantly distinguished Clade A from Clade C, and the second PC distinguished Clade A from some accessions in Clade D. These findings indicate that population genetics research might use analyses based on the chloroplast genome.

STRUCTURE, which calculates individual ancestry and admixture proportions assuming K populations, was used to examine population structure among the 104 accessions based on the entire chloroplast genome. We examined population structures with values of K between 2 and 10, with 10 iterations performed for each. The most suitable “blood lineages” of *P. montana* accessions were determined using the DeltaK, and the largest value was K = 4 (Figure 3B). The population structures with K = 3, 5, and 6 are shown in Figure S4.

### 2.5. Pueraria Montana Divergence Time

**Figure 4:**
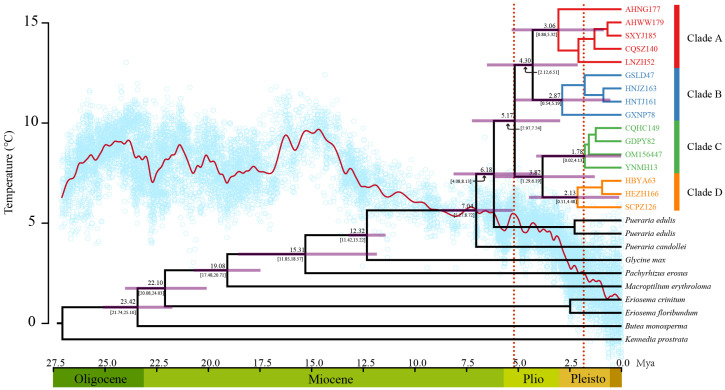
Glycininae divergence time. Numbers at nodes refer to their mean age (Ma). The purple bars correspond to the 95% HPD. A global average δ18O curve derived from benthic foraminifera, which mirrors the major global temperature trends about 28 MYA (red line), is shown below the tree [46,47].

The divergence time estimates for the stem and crown nodes of Pueraria s. str. were 12.32 million years ago (Ma) (95% highest posterior density [HPD]: 11.42–13.22 Ma) in the middle Miocene and 7.04 Ma (95% HPD: 5.72–8.72 Ma) in the late Miocene, respectively (Figure 4). Chloroplast genome-based phylogenetic inferences subdivided the 104 accessions into four clades. Molecular dating analyses suggested that they first diverged during the early Pliocene, around 5.17 Ma (95% HPD: 2.97–7.24 Ma). The crown age of Clades A and B was 4.3 Ma (95% HPD: 2.12–6.51 Ma) in the middle Pliocene. The divergence time between Clades C and D was 3.82 Ma in the middle Pliocene. The crown ages of Clades A, B, C, and D were 3.06, 2.87, 1.78, and 2.13 Ma, respectively, indicating that most genetic variants arose during the Pleistocene. The divergence time of the four clades coincided with global cooling during the Pliocene (Figure 4).

### 2.6. Genetic Evolution in Different Groups

**Figure 5:**
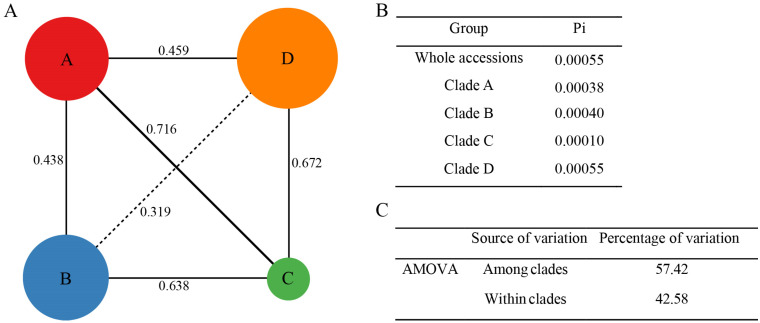
Chloroplast genome nucleotide diversity (π) and genetic distance (Fst). (**A**) The π (circle size) of the four clades (circles) and Fst among them. (**B**) Average π for the entire collection and each clade. (**C**) The AMOVA results among and within clades.

The four subclades identified in the phylogeny and PCA analyses were used to compare intra-clade divergence. The mean π of the four subclades were calculated as 0.00038, 0.00040, 0.00010, and 0.00055, respectively (Figure 5A,B). Clade A, comprising 464 SNPs and 190 indels, had the highest diversity, and Clade C, comprising 221 SNPs and 104 indels, had the lowest (Table 1).

Genetic distances (Fst) between the four subclades were 0.319–0.716, indicating high genetic differences among them (Figure 5A). Clade C had the highest genetic difference with Clades A and D. Clade C mainly included the var. *thomsonii* accession from GenBank. Four clades and the entire collection showed relatively high divergences. An analysis of molecular variance (AMOVA) suggested that both variations contributed to inter- and intra-clade differences (Figure 5C).

## 3. Discussion

### 3.1. Highly Variable Pueraria Montana Chloroplast Genome Sequences

Compared with nuclear DNA, the chloroplast genome had a lower substitution rate, occurring mostly at the species level or above. Therefore, detecting useful intraspecies polymorphisms is difficult in practice [23,48,49]. However, with the advent of NGS methods, many species have had more than two individuals sequenced, significantly improving our ability to assess mutation rates or detect intraspecies variations. Previous studies have assessed intraspecies mutations in chloroplast genome sequences, such as the medicine plants *Arnebia euchroma* and *Arnebia guttata* [50], the model grass plant *Brachypodium distachyon* [51], the endangered species *Bretschneidera sinensis* [16], the tertiary relict tree *Ginkgo biloba* [52], and cultivated rice [27]. These results indicate that the chloroplast genome sequences contain many mutations for evolutionary research.

This study analyzed the chloroplast genomes of 104 *P. montana* accessions, identifying 1118 SNPs and 556 indel variations with high intraspecies variability (Table 1). *P. montana* chloroplast genome showed higher variability compared with other published plant species, such as *Ginkgo biloba* (135 SNPs, 71 accessions) [52], Brachypodium distachyon (298 SNPs, 53 accessions) [51], soybean (44 SNPs, 2580 accessions) [53], *Coptis chinensis* (111 SNPs, 227 accessions) [19], and *Ulmus laevis* (32 SNPs, 54 accessions). Mutation heterogeneity across different plant lineages or species has been previously examined. There are two hypotheses invoking the mutation rates. The first hypothesis is that mutation rates are negatively correlated with generation times. This hypothesis suggests that long-lived plants will have lower mutation rates than short-lived species, such as herbaceous plants, due to their longer generation time. *P. montana*’s high intraspecific mutation rate may be partially explained by generation time since it is a perennial, semi-woody, climbing legume species with a middle to long generation time.

The second hypothesis is that long divergence times enable more mutations to accumulate. Estimated divergence times showed that *P. montana* evolved very early, around 5.17 Ma in the early Pliocene (Figure 4). Therefore, the persistence of genetically distinct populations through periods of historical climate variation during its long evolutionary history is the likely cause of its relatively high genetic diversity. The Northeastern and Southeastern Tibet Plateau’s uplift from the Pliocene to the early Pleistocene impacted Eastern China [54], with the East Asian summer monsoon [55] and South Asian summer monsoon [56] intensifying during that period.

Another possible reason for *P. montana*’s high mutation rate may be related to its type of chloroplast inheritance. Approximately 20% of angiosperm species have biparental plastid inheritance [57,58], which is associated with chloroplast genome rearrangement events and accelerated mutation rates. Some species in the Papilionoideae group have shown that the type of chloroplast inheritance is biparental [57,58], and its chloroplast genome structure has a large inversion (~56 kb) and gene and intron loss. In addition, the chloroplast genome has a high mutation rate [59,60,61]. Aberrant DNA repair, recombination, and replication with biparental inheritance may underlie increased substitution rates in the chloroplast genome [60,62].

Mutation heterogeneity also occurs in different regions of the chloroplast genome, such as mutation hotspots, with higher mutation rates. The IR regions are known to be more conserved than LSC and SSC regions. Mutations in the *P. montana* chloroplast genomes were found mostly in the LSC and SSC regions. The spacers psbZ-trnS and ccsA-ndhD had the highest marker variability in *P. montana* (Figure S1), and these markers are suited for investigating genetic diversity and population or subpopulation structure.

### 3.2. Pueraria Montana Genetic Divergence and Diversity

The *P. montana* complex includes three species (*P. montana*, *Pueraria lobata*, and *Pueraria thomsonii*) or three varieties (var. *montana*, *lobata*, and *thomsonii*). Phylogenetic evidence indicates that the *P. montana* complex forms a monophyletic group [29]. It is challenging to morphologically distinguish between species or varieties in the *P. montana* complex because characteristics used to differentiate taxa in dichotomous keys appear continuous and overlapping across its range, particularly for vars. lobata and thomsonii [28,63]. In this study, the monophyletic group of each species or variety was also not supported using the chloroplast genome data (Figure 2). The phylogeny of rDNA ITS data showed a lack of resolution (Figure S5) but still could be clustered into three clades by three well supported nodes (Table S1). In general, the varieties of *Pueraria* appear polyphyletic based on the results. Alternatively, the samples obtained from public databases could be by named incorrectly because of small differences between varieties. A previous study also supported a close relationship in nuclear sequence data between *P. montana* var. *lobata* and *P. montana* var. *thomsonii*, which were intermixed, suggesting their possible hybridization [36]. Nuclear SNP data has also indicated a higher potential for hybridization or introgression among the three varieties [30]. Hybridization or introgression may blur species boundaries and influence population structure.

*P. montana* genetic diversity has been assessed with various molecular markers. For example, an analysis of 13 allozymes across 1000 US accessions concluded that *P. montana* var. *lobata* possessed considerable genetic variation and lacked geographic structuring [33]. Analyses using RAPD [34], ISSR [36,37,38], and SSR [39,40] markers discovered higher genetic diversity in *P. montana* populations. Chloroplast genome dataset discovered *P. montana* with high diversity levels and population structure analyses identified four *P. montana* clades (Figure 2). *P. montana* is widely distributed in north-central, south-central, and southeast of China. The samples of each four clades did not reflect geographical distribution, indicating that geographic distance does not explain the genetic structure patterns.

In the context of its high ornamental, horticultural, agricultural, and ecological interest, *P. montana* was likely to migrate from multiple sources multiple times [36,39,41]. *P. montana* genetic studies have repeatedly revealed a high degree of variation among introduced populations, such as those found in the United States [32,34,36,64]. High genetic diversity levels in introduced populations may indicate multiple introductions [65]. Bringing such genetic diversity into proximity with untried environments that enable gene flow may also allow for novel adaptations, the consequences of which may have played important roles in *P. montana*’s invasiveness. However, the long-lived *P. montana* with high genetic diversity may reduce the likelihood of adaptive evolution due to its relatively small effective number of generations [30].

## 4. Materials and Methods

### 4.1. Samples and Chloroplast Genome Sequencing

We collected 70 *P. montana* samples from China, covering most of its wild distribution in China (Table S1). The 70 samples (accessions) were collected from more than 19 provinces, including Anhui, Chongqing, Gansu, Guangdong, Guangxi, Guizhou, Hebei, Henan, Hubei, Hunan, Jiangsu, Jilin, Liaoning, Shaanxi, Shandong, Shanxi, Sichuan, Yunnan, and Zhejiang (Table S1). Owing to the overlapping taxonomic characteristics, we did not distinguish the three varieties in this study. For each accession, young leaves were sampled and dried with silica gel. Voucher specimens were deposited in the herbarium of the National Resource Center for Chinese Materia Medica at the China Academy of Chinese Medical Sciences. Total genomic DNA was extracted using the modified CTAB (mCTAB) method, and DNA quality and quantity were assessed by agarose gel electrophoresis (1% *w*/*w*).

Total DNA was fragmented into 350 bp fragments by sonication. Illumina paired-end DNA libraries were constructed using NEB Next Ultra DNA Library Prep Kit for Illumina (NEB, USA). Each sample was barcoded with a unique index, and libraries were pooled for sequencing on an Illumina HiSeq X-Ten platform at Novogene (Tianjin, China). Each accession yielded ~10 Gb of 150-bp paired-end reads.

The raw data was subject to quality control using Trimmomatic 0.36 [66] with the following parameters: LEADING, 20; TRAILING, 20; SLIDING WINDOW, 4:15; MIN LEN, 36; and AVG QUAL, 20. The cleaned data were used to assemble the chloroplast genome and rDNA ITS using GetOrganelle [67] with 85, 95, and 105 kmer lengths. Where GetOrganelle failed to assemble the complete chloroplast genome, we assembled it following the methods of Dong et al. [5]. The Perl script Plann [68] was used to annotate the chloroplast genome using the published *P. montana* var. *lobata* genome (GenBank accession number: MT818508) as the reference sequence. The annotated chloroplast genome sequences are deposited in the GenBank, accession number OP963859- OP963928.

### 4.2. Variation Identification and Statistics

The chloroplast genomes of 70 *P. montana* accessions and 44 downloaded from GenBank were aligned using MAFFT version 7.490 [69]. Se-Al version 2.0 [70] was used to manually correct alignment errors, such as polymeric repeat structures and small inversions. SNPs and indels were identified over the entire chloroplast genome, and genetic distances were calculated among accessions. The numbers and distributions of SNPs and indels were summarized using MEGA version 7.0 [71] and DnaSP version 6 [72].

### 4.3. Population Structure and Principal Component Analysis

The accessions’ population structures were investigated using STRUCTURE [73]. The optimal cluster number (K) was determined by running the K-means clustering algorithm from K = 2 to K = 10 with ten runs for each K. The STRUCTURE workflow was according to the previous study (Wang et al., 2022c). A principal component analysis (PCA) was performed using PLINK [74] to evaluate the genetic structure. The R v4.1 statistical software’s ggbiplot package was used to plot the graphs [75].

The complete chloroplast genomes of 104 *P. montana* accessions were aligned with those for *Pueraria edulis* and *Pueraria candollei* var. *mirifica* as the outgroups using MAFFT version 7 [69]. Two methods, maximum likelihood (ML) and Bayesian inference (BI), were used to reconstruct the phylogenetic tree for the *P. montana* accessions. The best-fit model for the ML analyses was selected by ModelFinder [76] based on the Akaike Information Criterion and BI analyses were based on the Bayesian information criterion. The ML analysis was performed in RAxML-NG [77], and support values were assessed by 1000 bootstrap replicates.

BI analysis was performed in Mrbayes v3.2 [78]. Four Markov chain Monte Carlo (MCMC) simulations were run from random trees for 10 million generations, with trees sampled every 1000 generations. The stationary phase was examined using Tracer 1.6 [79], and the first 25% of the sampled trees were discarded as “burn-in.” The majority-rule consensus tree was created using the remaining trees and estimated posterior probabilities. All trees were visualized using FigTree version 1.4 (http://tree.bio.ed.ac.uk/software/figtree/, accessed on 21 April 2023).

### 4.4. Genetic Diversity in P. montana Subclades

Nucleotide diversity (π) and subclade divergence (fixation index [Fst]) were calculated using the whole chloroplast genome dataset to assess genetic diversity among the different clades. The network of all samples was constructed. Haplotype data were analyzed with DnaSP version 6 [72], and a median-joining network was constructed using PopArt version 1.7 [80,81].

### 4.5. Divergence Time Estimation and Profiling

We used the chloroplast genome to estimate the divergence times of the different subclades. This dataset included 16 *P. montana* accessions from different subclades and nine Glycininae species as the outgroups. According to the average values of Egan et al. [29] in a calibrated analysis, we used four secondary priors for this study: (1) the crown age of the core Phaseoleae was set to 23.52 million years ago (Ma); (2) the Phaseolinae stem age was set to 19.26 Ma; (3) the crown node of the Pueraria s. str. and Glycine was 12.71 Ma; and (4) the Pueraria s. str. crown age was 7.85 Ma. All four secondary priors were placed under normal distribution with a standard deviation of 1.

The divergence time analyses were performed using BEAST 2 [82]. We chose a general time reversible model and a relaxed molecular clock model with an uncorrelated lognormal distribution. We ran 500,000,000 generations of the MCMC algorithm, with samples taken every 50,000 generations. Effective sampling sizes > 200 were used to measure convergence using Tracer version 1.6 [79]. A maximum clade credibility tree with mean heights was built in TreeAnnotator after discarding the first 10% of the trees as burn-in.

## 5. Conclusions

This study used whole chloroplast genomes to examine *P. montana*’s genetic diversity, identifying genome-wide variability and contributing to a better understanding of the divergence history among its populations. The phylogenetic, PCA, and structure analyses showed genetic differentiation and divergence among *P. montana* populations. Chloroplast genome sequences showed high variability and provided novel insights into evolutionary history.

## Figures and Tables

**Table 1:** Summary of the total variations (SNPs/indels) detected in the whole collection and each clade.

Group	Accessions	All Variations			
		SNPs	Indels	Total	Density/kb
Clade A	28	464	190	654	4.09
Clade B	26	422	225	647	4.04
Clade C	39	221	104	325	2.03
Clade D	11	232	151	383	2.39
Total	104	1118	556	1674	10.46

## Data Availability

The data is contained within the manuscript and Supplementary Materials. The chloroplast genome sequences under this study are deposited in the GenBank, accession number OP963859- OP963928.

## Supplementary Materials

The following supporting information can be downloaded at: https://www.mdpi.com/article/10.3390/plants12122231/s1, Figure S1. Nucleotide diversity (π) in *P. montana*’s chloroplast genome based on a window size of 800 bp and a step size of 100 bp. Figure S2. Network of all 104 *P. montana* accessions. Figure S3. The delta K results of STRUCTURE analysis. Figure S4. Population structure of all 104 accessions with K = 3, 5, and 6. Figure S5. The ITS phylogenetic tree of 70 *P. montana* collected accessions. Table S1. Detail information of 107 sequenced or downloaded chloroplast genomes.
