# Supplementary material for: Pueraria montana Population Structure and Genetic Diversity Based on Chloroplast Genome Data
Source: Plants (Basel). 2023 Jun 6;12(12):2231. doi: 10.3390/plants12122231 (PMC10302751; doi:10.3390/plants12122231)
Supplement: Supplementary file 1 [file plants-12-02231-s001.zip › plants-2385423 Supplementary Figures.pdf]

Supplementary Materials

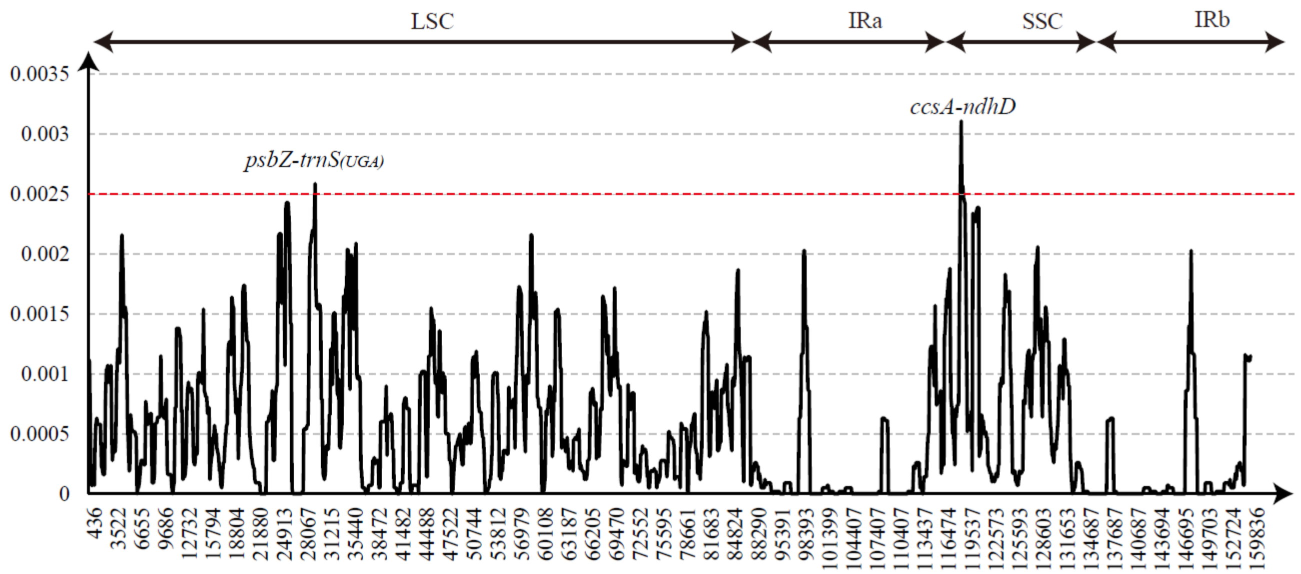

Figure S1. Nucleotide diversity (π) in *P. montana*'s chloroplast genome based on a window size of 800 bp and a step size of 100 bp.

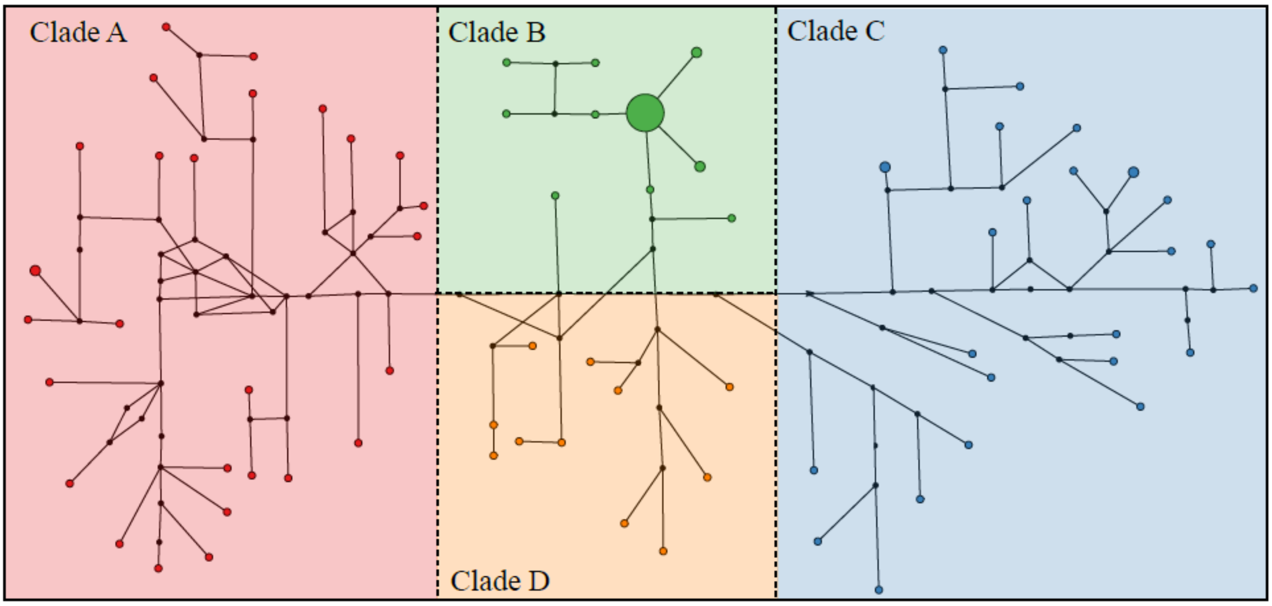

Figure S2. Network of all 104 *P. montana* accessions.

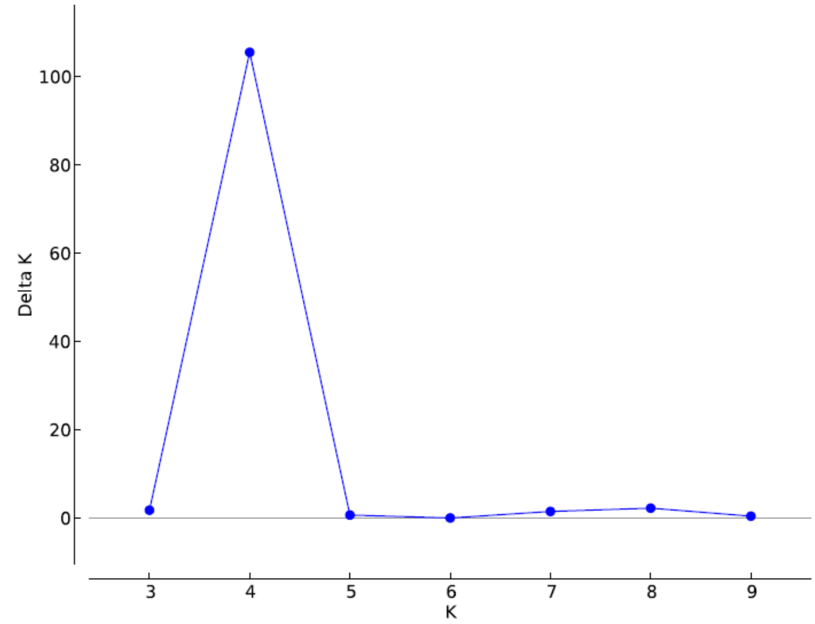

Figure S3. The delta K results of STRUCTURE analysis.

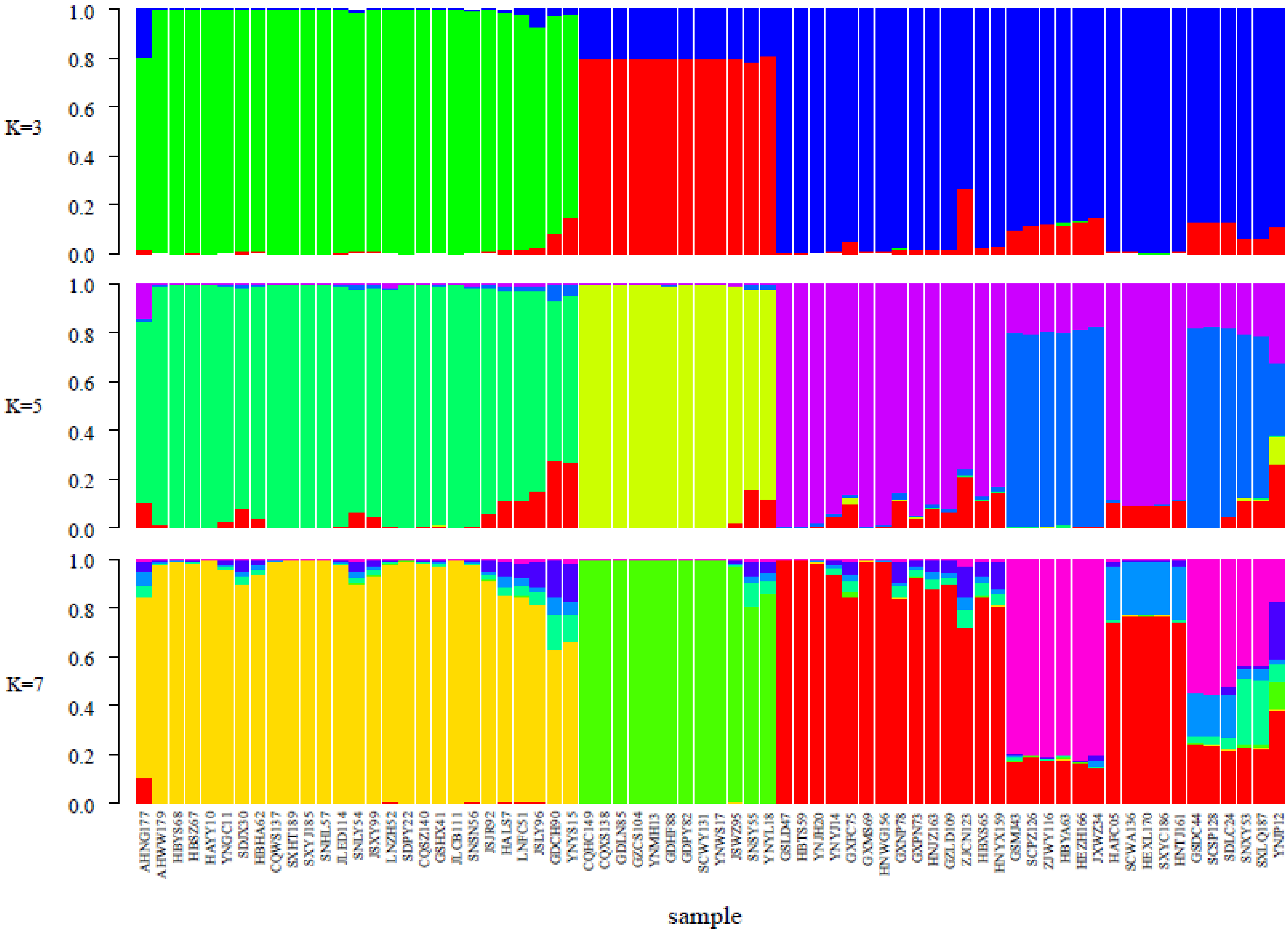

Figure S4. Population structure of all 104 accessions with K = 3, 5, and 6.

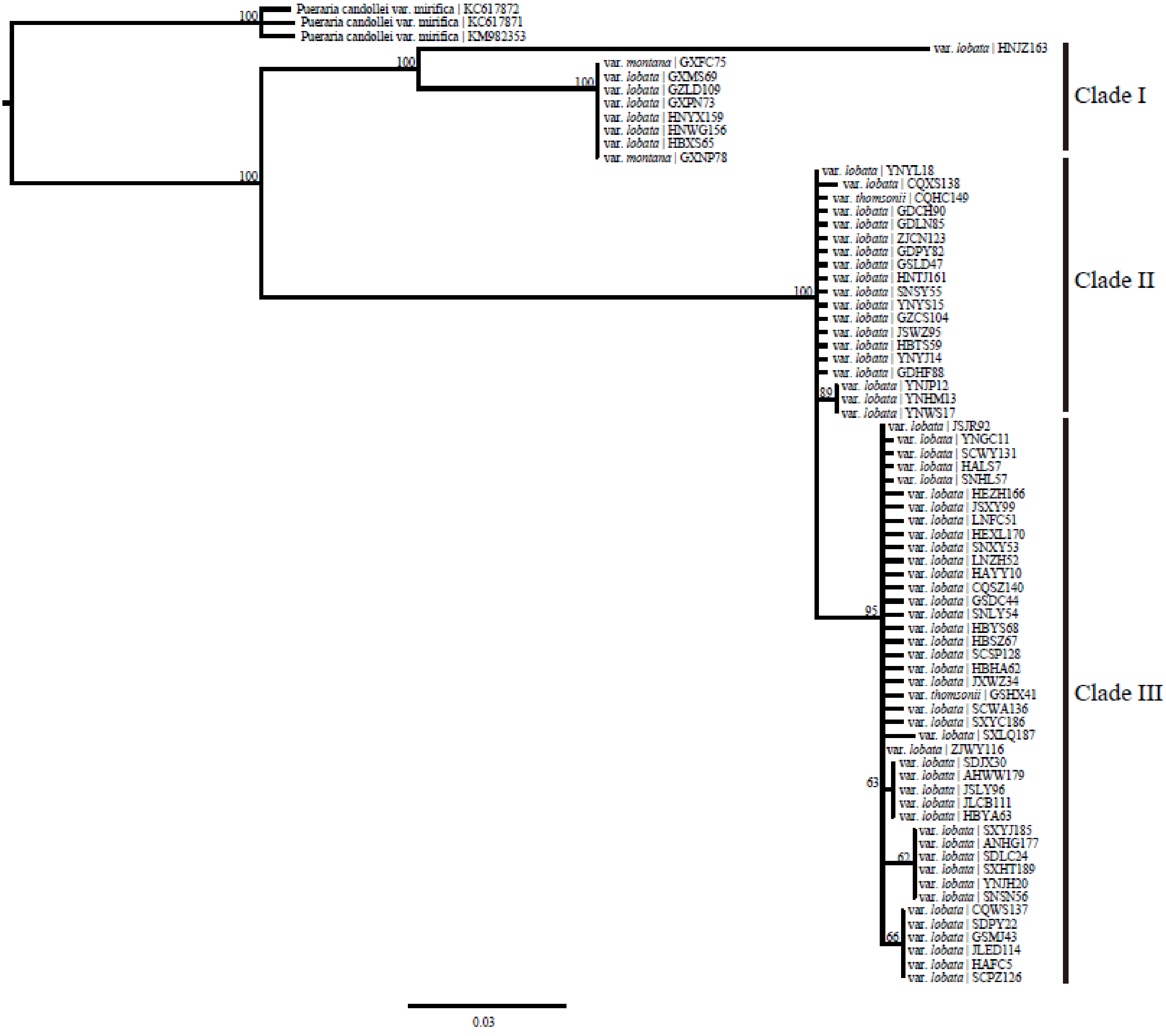

Figure S5. The ITS phylogenetic tree of 70 *P. montana* collected accessions.
